# The potential role of silver nanoparticles in the tongue wound healing

**DOI:** 10.1186/s12903-025-07547-1

**Published:** 2026-01-21

**Authors:** Kholoud Moustafa ElSherbiny, Huda Rizq A. Elnaby, Mona H. El-Zekrid, Nessma Sultan

**Affiliations:** 1 Oral Biology department, faculty of dentistry, Kafr elsheikh university, Kafr elsheikh, Egypt; 2 Faculty of dentistry, New Mansoura University, Mansoura, Egypt; 3 Oral Biology department, faculty of dentistry, Mansoura University, Mansoura, Egypt; 4 Oral Biology and dental morphology department, faculty of dentistry, Mansoura National University, Gamasa, Egypt

**Keywords:** Silver nanoparticles, ADSCs, Tongue ulcer, Wound healing

## Abstract

**Objectives:**

The tongue is a complex muscular organ that can be affected by chronic ulcers and malignant tumors, which need proper management to improve healing and tissue regeneration. Hence, we aimed to assess the efficiency of adipose tissue stem cells (ADSCs) treated with/without silver nanoparticles (AgNPs) on tongue wound healing.

**Materials and methods:**

Fifty-six male white albino rats were divided into four groups; group I: tongue defects were prepared on the tongue dorsal surfaces using a tissue punch rotary drill for standardization, and were left untreated, group II: tongue defects were treated subepithelial using 2.5 µg/ml AgNPs solution using insulin syringe on the same day of defect preparation, group III: tongue defects were injected with 200 µl of ADSCs suspension (3.5 × 10^5^ cells) and group IV: tongue defects were injected with 200 µl of ADSCs previously treated with 2.5 µg/ml AgNPs solution. Four- and seven-day follow-up following treatment, tongue specimens were collected, and histological assessment was conducted by hematoxylin and eosin (H&E) stain and immunohistochemical assessment of anti-TNF-alpha and anti-TGF-β, followed by histomorphometric assessment. Two-way ANOVA was utilized to compare between the groups after 4 and 7 days.

**Results:**

The treated groups revealed a significant improvement characterized histologically by regenerated tissue with well-developed, thick-walled, and well-organized vessels and a significant decrease in defect depth in comparison with the control group. Group IV displayed a significant reduction in TNF-alpha and a significant increase in TGF-β antibodies, denoting its anti-inflammatory and neovascularization actions correspondingly.

**Conclusion:**

ADSCs treated with AgNPs could significantly enhance regeneration and filling of tongue defects by decreasing tissue inflammation and improving neovascularization, being better than using ADSCs or AgNPs alone. Therefore, it may be a possible treatment plan for enhancing the healing of tongue ulcers.

## Introduction

The tongue is a muscular organ that can be damaged by several illnesses and tumors, which include squamous cell carcinoma, granular cell tumor, and erythroplakia, which require surgical removal of the affected site [1]. Numerous factors may contribute to mouth ulcers that also impact the tongue and are classified as acute and chronic based on their traits [2].

Antibiotics, analgesics, anti-inflammatory drugs, angiogenic factors, herbal remedies, and specialized localized therapies, including chemical cauterization, surgical excision, and laser therapy, can all be used to treat oral and maxillofacial issues. However, these treatments might not make much of a difference or could have a lot of unintended harmful effects [3, 4]. Oral diseases are treated using the conventional drug-delivery method, which primarily comprises tablets and gels, to manage the defect by applying the proper biomaterial [5].

The broad scientific field of nanotechnology has attracted the attention of many scientists and business experts as it provides the simple production of metal-based biocompatible nanoparticles. Biomaterials containing silver (Ag) and silver nanoparticles (AgNPs) are increasingly being utilized as wound dressings in both clinical and experimental contexts. Since the introduction of Ag sulphadiazine, an antimicrobial-antibiotic combination that provides a broad-spectrum and effective therapy, Ag-based compounds have been utilized to treat wounds since the early 1970s. Basic dressings were enhanced with various forms of Ag to strengthen their antibacterial properties and ward against infections as the majority of non-healing wounds include bacterial colonization and biofilm formation [6].

Since adipose tissue stem cells (ADSCs) are frequently a result of numerous medical and cosmetic treatments, they are the most widely used source of mesenchymal stem cells (MSCs) owing to their good accessibility and ease of acquisition. ADSCs have a spindle shape and a rapid rate of multiplication and meet the minimal requirements of MSCs since they can develop into adipocytes, chondrocytes, and osteoblasts [7]. Compared to other MSCs sources, ADSCs generate and release a greater number of angiogenic factors, which include IGF-1, TGF-β1, VEGF, angiogenin, interleukin 8, growth-regulated protein, and platelet-derived growth factor. It has been established that angiogenic factors exist at the mRNA and protein levels, both in extracellular vesicles and in a soluble form in a conditioned medium [8].

According to earlier research, MSCs can absorb AgNPs by macropinocytosis and clathrin-dependent endocytosis, and after these nanoparticles are taken up, Ag agglomerates develop in the cytoplasm. Concluding that the biological impacts of MSCs on different cells are caused mainly by dissolved Ag ions [9]. Thus, in this context, we discussed first the cytotoxic effect of different concentrations of AgNPs on ADSCs then, the healing potential and anti-inflammatory effects of ADSCs treated with or without AgNPs was assessed in comparison to using AgNPs as a sole treatment material.

## Materials and methods

Sixty healthy male white albino rats (200–250 g) were utilized. The entire in vitro and in vivo experimental approaches were conducted at Nile Experimental Research Center for Experimental Research at the animal house unit under approved rules of the ethical committee, Faculty of Dentistry, Kafr El Sheikh University, Egypt, with KFS-IACUC/293/2025. Rats were group-housed with optimized humidity and temperature and a twelve-hour light/dark cycle. Rats received a conventional laboratory diet and were permitted access to drinking water.

### Sample size calculation

A total of sixty rats were utilized in this experiment; fifty-six were divided into four groups/14 rats each, and 4 rats were utilized for the isolation of ADSCs. Two-way ANOVA was utilized to analyze variance; according to Cohen, fourteen samples per group/seven samples per time point were adequate to determine the effect size of 0.89 at a power (1-β = 0.9) of 80% (*P* < 0.05). Sample size underwent calculation using G*Power V 3.1.9.6 (Cohen), in which f is the effect size = 0.89; alpha = 0.05; beta = 0.1; Power = 1-β = 0.9. Sample size was calculated and considered adequate based on the previous study, Ata et al., [10].

### Research objectives

RQ1: In comparison to ADSCs, how do AgNPs affect the quality of tongue wound healing?

*Rationale: For oral surgery and trauma management*,* it is crucial to comprehend whether AgNPs can hasten or enhance healing.*

RQ2: Does the use of AgNPs in tongue wound healing models have any adverse effects or cytotoxic results? How do they compare to the treatment of ADSCs?

*Rationale: Before proposing AgNPs for oral applications where mucosa sensitivity may be higher*,* safety evaluation is crucial.*

RQ3: How do AgNPs affect important biological processes, including inflammation, and epithelialization that are involved in the healing of tongue wounds?

*Rationale: Determining the stage of healing that AgNPs alter aids in elucidating their safety profile and mechanistic function.*

### Isolation of ADSCs

After separating the adipose tissue that surrounds the epididymis, they were put in a petri dish (Sterilin, Fisher scientific, UK) with phosphate-buffered saline (PBS). A knife and forceps were used to chop these fat pads into small pieces in the biosafety cabinet. These sliced pieces of adipose tissue were placed in a 50 mL centrifuge tube with 10 mL of sterile PBS.

The harvested adipose tissue was first positioned in a sterilized petri dish, and the final tissue weight was detected. After that, the tissue was gently crushed into minor fragments by using a scalpel or scissors. Adipose tissue specimens were positioned in a 15 mL tube and washed 4 times by using a similar volume of PBS containing 3% penicillin/streptomycin solution till the supernatant was totally clear. After that, the adipose solution was transferred to a 50 mL tube, and an equal volume of 0.1% type I collagenase (Merck, Millipore, UK) solution was added. Specimens were, as a result, incubated for 1 h in a 37 °C. Collagenase activity was stopped by adding complete growth media; Dulbecco’s modified eagles’ medium (DMEM, Biosera, UK) + 20% fetal bovine serum (FBS, Life science, UK) then centrifuging at 2000 r.p.m for 5 min. The supernatant was removed; the pellet was re-suspended in PBS, passed across a cell strainer (70 μm) (Greiner Bio-One International, Kremsmünster, Austria) and centrifuged at 2000 r.p.m for 5 min. The supernatant was aspirated, and the cell pellet was re-suspended in DMEM supplemented with 20% FBS, 100 µg/mL L-glutamine (Life science, UK), and 100 µg/mL antibiotic-antimycotic solution (Biowest, USA). Two days following plating, aspiration of the medium was performed, and fresh complete culture medium was added [11].

### Characterization of ADSCs

After being fixed for 15 min with 4% paraformaldehyde, ADSCs were washed in PBS and then labelled for 60 min with primary antibodies, CD90, CD105, CD34, and CD14. After that, cells were labelled for 45 min using secondary antibodies conjugated with fluorescein isothiocyanate. Using FACS Calibur flow cytometry (BD Immunocytometry Systems), the presence of cells that stained negative for CD34 and CD14 and positive for CD90 and CD105 was assessed.

### Culturing with sacrofere [12]

Labeling of stem cells for their localization in the epithelium or underlying lamina propria was done using iron sucrose (sacrofere) (Amoun Company, Cairo, Egypt). 20 µl/ml iron sucrose was added into the cell culture media for 120 min prior to injection. Prussian blue stain was applied to determine the iron oxide-labeled cells.

### Preparation of AgNPs

The chemical reduction approach was used to prepare AgNPs. AgNO_3_ (Merck, Germany) solution was utilized as Ag^1+^ ions precursor. The polyvinylpyrrolidone was utilized as a stabilizing agent, and sodium borohydride (Loba-India) as a reducing agent. The color of the solution gradually changed into grayish yellow, which indicates the conversion of the Ag^1+^ ions into AgNPs.

### Characterization of AgNPs

#### Optical properties

 UV-Vis absorption spectra were attained on Cary series UV-Vis- NIR, Australia.

#### Size and shape

 Electron microscopes use electrons that have short wavelengths and, as a result, permit assessment of matters with atomic resolution. In such a setting, TEM studies were carried out on JEOL JEM-2100 h-TEM at an accelerating voltage of 200 kV. To prepare samples for TEM, a drop of colloid suspension was placed on a Formvar carbon-coated, 300-mesh copper grid in the respective solvent. The samples were then allowed to evaporate in air at ambient conditions. An image analysis software package was used to detect the size distribution and the average size.

### Cytotoxicity of AgNPs on ADSCs [13]

The cytotoxic effect of AgNPs was assessed using the MTT assay (MTT cell growth kit; Chemicon, Rosemont, IL). ADSCs (4000 cells/well) were cultivated in 96 well plate in DMEM + 20% FBS for 24 h. After that, different concentrations (2.5 µg/ml, 5 µg/ml, 10 µg/ml, 15 µg/ml, 20 µg/ml) of AgNPs were added to the culture medium. Three days following incubation with AgNPs, 1 mg/ml of MTT was added, and the cells were incubated for 4 h. After that, 100 µl of DMSO was added to form formazan. The absorbance at 570 nm was measured utilizing a microplate reader (ELx 800; Bio-Teck instruments, Winooski, VT) and 690 nm as a reference wavelength. All conditions were analyzed in triplicate separately. The following equation was used to quantify the results as a percentage of viability after the values from each well were standardized against the control group (cells with medium only): Cell viability % = Absorbance of test ÷ Absorbance of control x100 [14].

### Tongue surgical defect

**Fig. 1:**
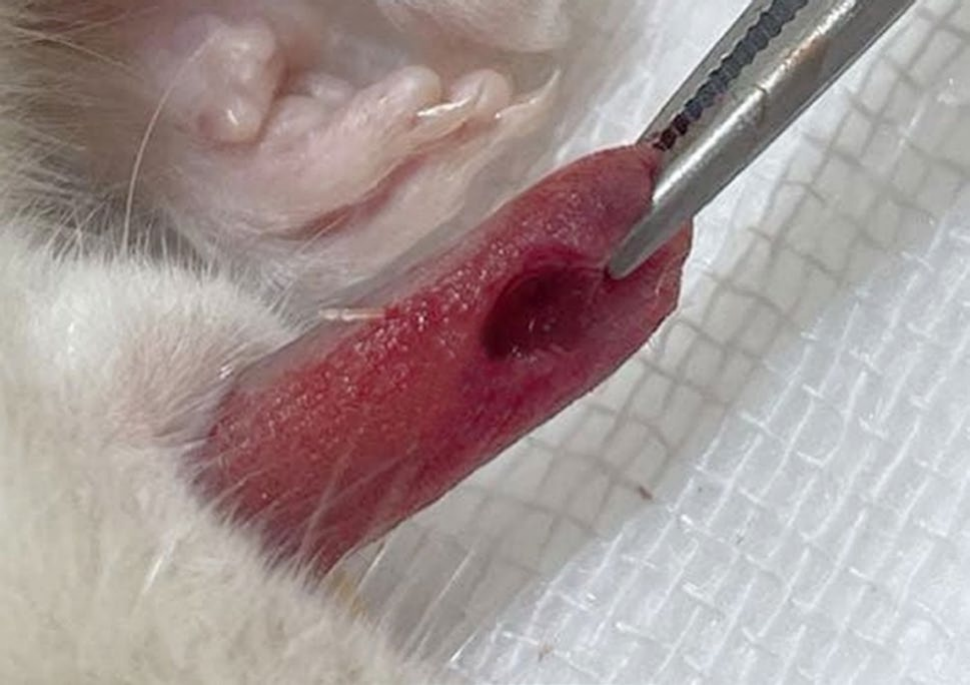
Showing the induced tongue defect

Rats were anaesthetized with intraperitoneal injection of xylazine (12.5 mg/kg) and ketamine hydrochloride (100 mg/kg) (ADWIA Co., Egypt) [15]. After securing anesthesia, the tongue was pulled out of the mouth with forceps **(**Fig. 1**)** and then disinfected with a sterile cotton pellet that was moistened. A sterile, rounded, vertical incision was performed by utilizing a four-mm rounded surgical punch and the scalpel blade number fifteen to excise residual tissues and create a sterile defect in the left side of the anterior dorsal tongue aspect at a depth of 3 mm into the muscle layers to cause injury to the epithelium and the stroma [16].

### Experimental protocol

#### Preparation of ADSCs suspension

When ADSCs reached confluence, the cells were counted using a hemacytometer, and 3.5 × 10⁵ cells were suspended in 200 µl of growth culture media DMEM and were injected into the defect area using an insulin syringe.

#### Grouping

Surgical interferences were conducted on all rats and were divided randomly into four groups (14 rats each); Group I: Rats served as the control group, and a surgical defect was performed on the left anterior tongue dorsal surface, and received no treatment. Group II: Dealing with rats was as in group I, and the defect area was injected subepithelially with 2.5 µg/ml of AgNPs solution using an insulin syringe [17]. Group III: Rats were treated as in group I, and the defect was injected with 200 µl of ADSCs suspension [18]. Group IV: Rats were treated as in group I, and the defect was injected with 200 µl of ADSCs suspension previously cultured/treated with 2.5 µg/ml AgNPs [17].

#### Postoperative care

Each rat then received 0.5 ml IM antibiotic amoxicillin (Emox, Epico, Egypt), 1gm/10 ml distilled water, 2.5 mg/kg intramuscular every 24 h flunixin-meglumine as an anti-inflammatory drug, and 0.2 ml IM ketoprofen 50 mg (Ketophan 50, European Egyptian Pharm, IND, Egypt), every 24 h for 4 days [15].

Seven rats from each group were sacrificed by utilizing halothane at each time point, after four and seven days. Following scarification, the tongue was cut using scissors from the angle of the mouth back between the jaws; after that, the lower jaw was pulled down to dislocate the jaws. After that, the tongue base was cautiously cut by using a scalpel by transection at the circumvallate papillae. The tongues were excised in a gentle manner by utilizing forceps; then washed and stored in 10% formalin saline, and were transferred for histologic and immunohistochemical (IHC) assessment.

### Histologic and immunohistochemistry (IHC) assessment

Preparation of paraffin blocks of tongue was conducted; after that, 4 μm serial specimens were cut by a microtome. Deparaffinization and rehydration were done, and after that, H&E staining was conducted for evaluation of alterations in defect healing progress, as well as tissue regeneration. With regard to IHC, H2O2 was utilized to block endogenous peroxidase, then antigens were regained by boiling in citrate buffer. Incubation of the slides were after that conducted by using primary antibody’s (Abs) for TNF-α (Rabbit recombinant multiclonal [A11534], cat,#ab283655, Cambridge, United Kingdom, dilution 1:100) as an inflammatory marker and TGF-β (Rabbit monoclonal, cat. # ab32152, United Kingdom, dilution 1:250) to evaluate neovascularization and tissue repair then incubation with the secondary biotinylated Ab, after that streptavidin biotin complex. The chromogen used was diaminobenzidine, after which a counterstaining with harris hematoxylin was performed [10].

### Digital image analysis

For image analysis, immunohistochemically stained slides were used. At each time point, seven tissue slides were made from both experimental and control animals. Four arbitrary regions in the center of the defect were photographed (at a magnification of 100×). So, 28 images from each group/time point were analyzed, with calculation of the percentage of positive brown staining area. VideoTest Morphology^®^, a Russian company that describes a specific built-in procedure for area and percentage area measurements, and an Intel^®^ Core I7^®^-based computer were used to analyze the images. Images utilized for assessment were stained by using TNF-alpha and TGF-β. The positive reaction appeared brownish in color. The data were expressed as the mean positive brown staining percentage ± SD.

### Statistical analysis

Data analysis was conducted by using GraphPad Prism 9. The normality of data was assessed by utilizing the Shapiro-Wilk test. Quantitative data were expressed as mean ± SD for normally distributed data. The level of significance was set at the 0.05 level. The Two-way ANOVA with Tukey Post-Hoc test was utilized to compare the means of three or more independent groups after 4 and 7 days.

## Results

### Flow-cytometric phenotypic characterization

**Fig. 2:**
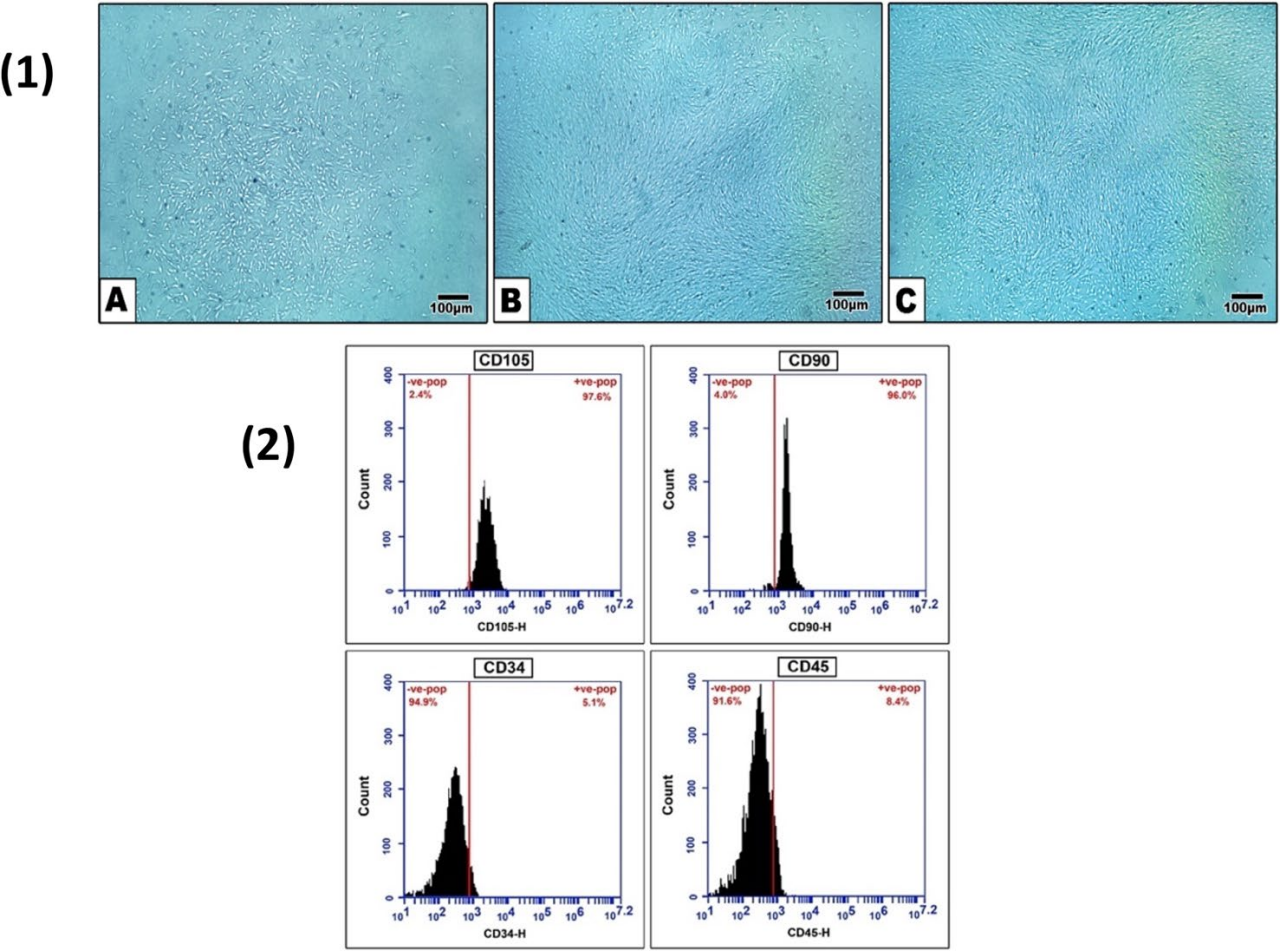
(1) Isolated ADSCs is depicted, where (**A**) at P0 displays colony of adherent cells, (**B**) At P1, the adherent cells exhibited spindle-like characteristics, however at P3 (**C**), the colonies began to expand, and a monolayer cell sheet is formed. (100 μm scale bar). (2) Flow cytometry charts display ADSCs phenotypic analysis. The histogram displays fluorescence intensity as a measuring variable (x-axis) and cell count (y-axis)

The 3rd passage ADSCs were exposed to cell surface phenotypic marker analysis, in which the MSCs markers CD105 (97.6%) and CD90 (96.0%) were demonstrated to be highly positive. In contrast, the hematopoietic markers CD45 (8.4%) and CD34 (5.1%) were negative, confirming the stemness of the isolated cells **(**Fig. 2**)**.

### MTT assay

**Fig. 3:**
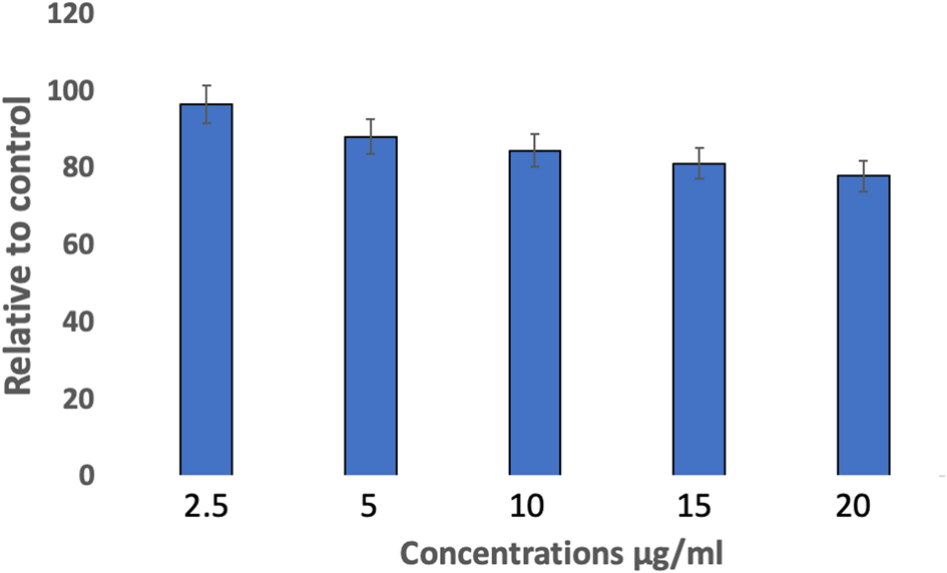
MTT analysis of viable ADSCs percentage cultured with AgNPs. ADSCs were subjected to complete medium (control), 2.5, 5, 10, 15 and 20 µg/ml of AgNPs for 72 h. 5, 10, 15 and 20 µg/ml showed significant difference in comparison to control (*P* < 0.05). Data are presented as mean±SD

MTT assay tested the viability of ADSCs seeded with various concentrations of AgNPs for 72 h **(**Fig. 3**).** ADSCs’ viability in 10, 15, and 20 µg/ml concentrations of AgNPs was significantly lower than that of the control (*P* < 0.01), suggesting their cytotoxicity. In contrast, at a concentration of 5 µg/ml, viability revealed a significant difference compared to healthy controls (*P* < 0.05), while showing borderline significance (*P* < 0.06) compared to the 2.5 µg/ml concentration. 2.5 µg/ml concentration showed a high percentage of viable cells, without showing a significant difference with controls. Thus, 2.5 µg/ml of AgNPs had been used in the following experiments.

### Characterization of AgNPs (Fig. 4)

**Fig. 4:**
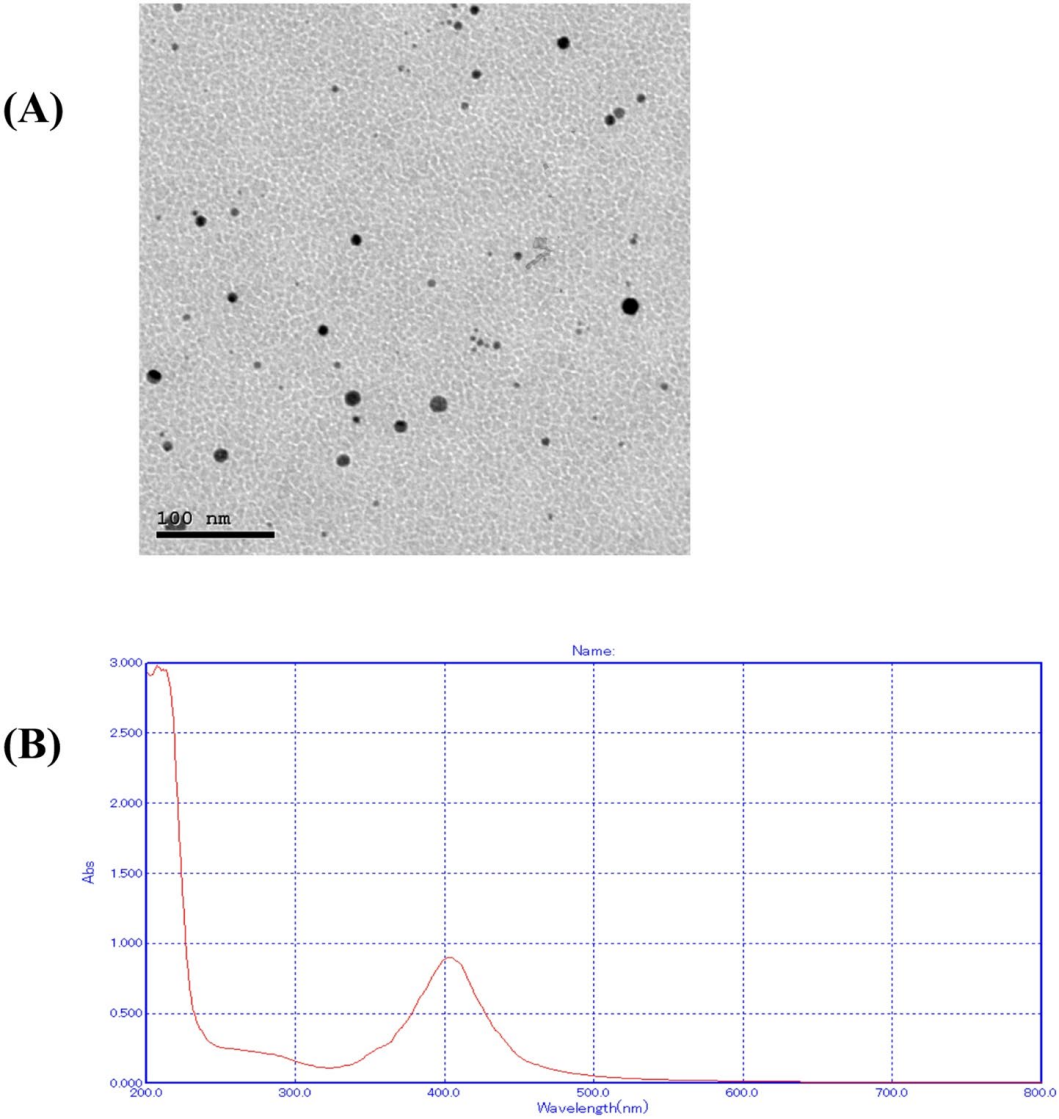
Characterisation of synthetized AgNPs. (**A**) TEM, (**B**) ultraviolet visible spectra (UV-Vis) of AgNPs sample

The chemical reduction approach was used to prepare AgNPs, leading to a concentration of 100 parts per million. The formed NPs were characterized by transmission electron microscope (TEM) and UV-Vis. Particle shape and size of the synthesized AgNPs were detected by TEM image analysis **(**Fig. 4A**)**. Based on the obtained TEM micrographs, AgNPs were spherical and well-separated from each other. The mean size of the AgNPs is established to be 25 ± 5 nm. The UV–Vis spectra of the synthesized NPs **(**Fig. 4B**)** verified their optical characteristics, with the LSPR peaks noticed at 410 nm for AgNPs, consistent with formerly recorded values for quasi-spherical NPs of identical size [19].

### Prussian blue stain (Fig. 5)

**Fig. 5:**
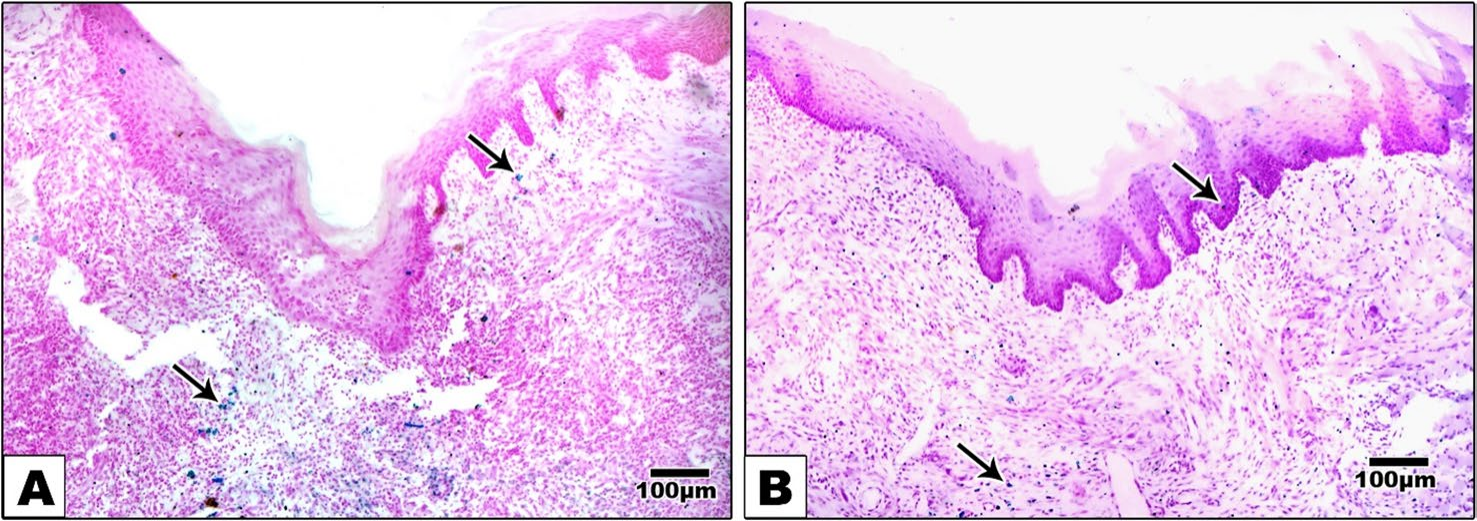
Experimental groups treated with ADSCs only (**A**) or combined with AgNPs (**B**) revealed certain iron oxide labelled stem cells seemed in defect area of tongue surface at entire stages as granular purple color. Cytoplasm displayed a pink colour, red nuclei and zones enclosing ferric iron revealed bright blue

Prussian blue staining of groups treated with either ADSCs only or ADSCs + AgNPs revealed that some iron oxide-labelled stem cells appeared in the defect region, revealing the role of ADSCs in the tongue healing process.

### Histological and immunohistochemical analysis: (Figures 6 and 7)

**Fig. 6:**
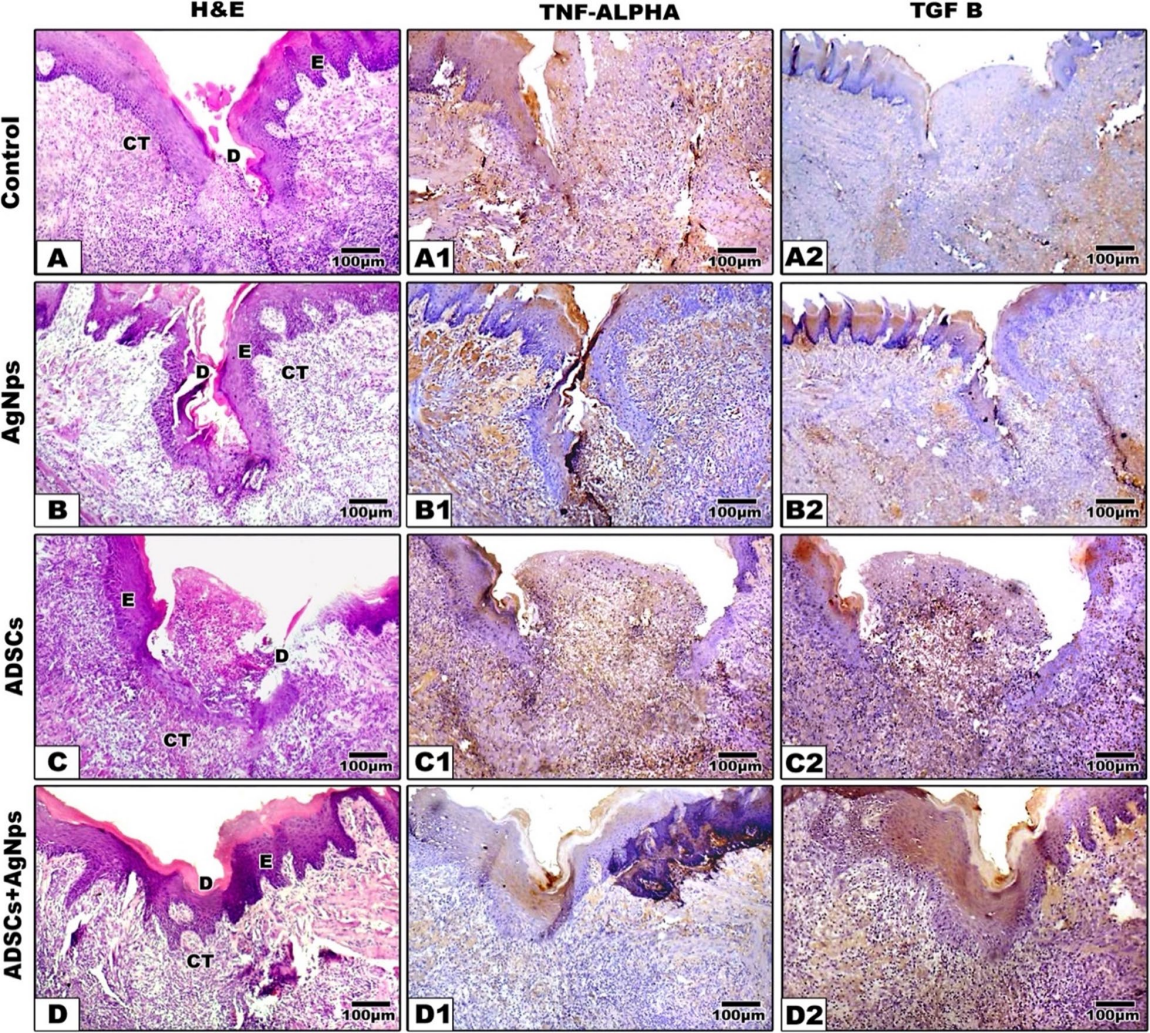
Photomicrographs showing Hematoxylin & Eosin (**A**-**D**), TNF- α (A1-D1) and TGF-β (A2-D2) expression in different treated groups after 4 days. In the controls, the defect filled with granulation tissue with intensive inflammation. In AgNPs, the defect started to close with extensive inflammatory infiltration. In ADSCs some defects were almost filled with granulation tissue with inflammatory cell infiltrate. While group IV (ADSCs + AgNPs) revealed closure of the tongue wounds with no visible tongue papillae. E: epithelium, CT: connective tissue, M: muscles, D: defect, scale bar = 100 μm

**Fig. 7:**
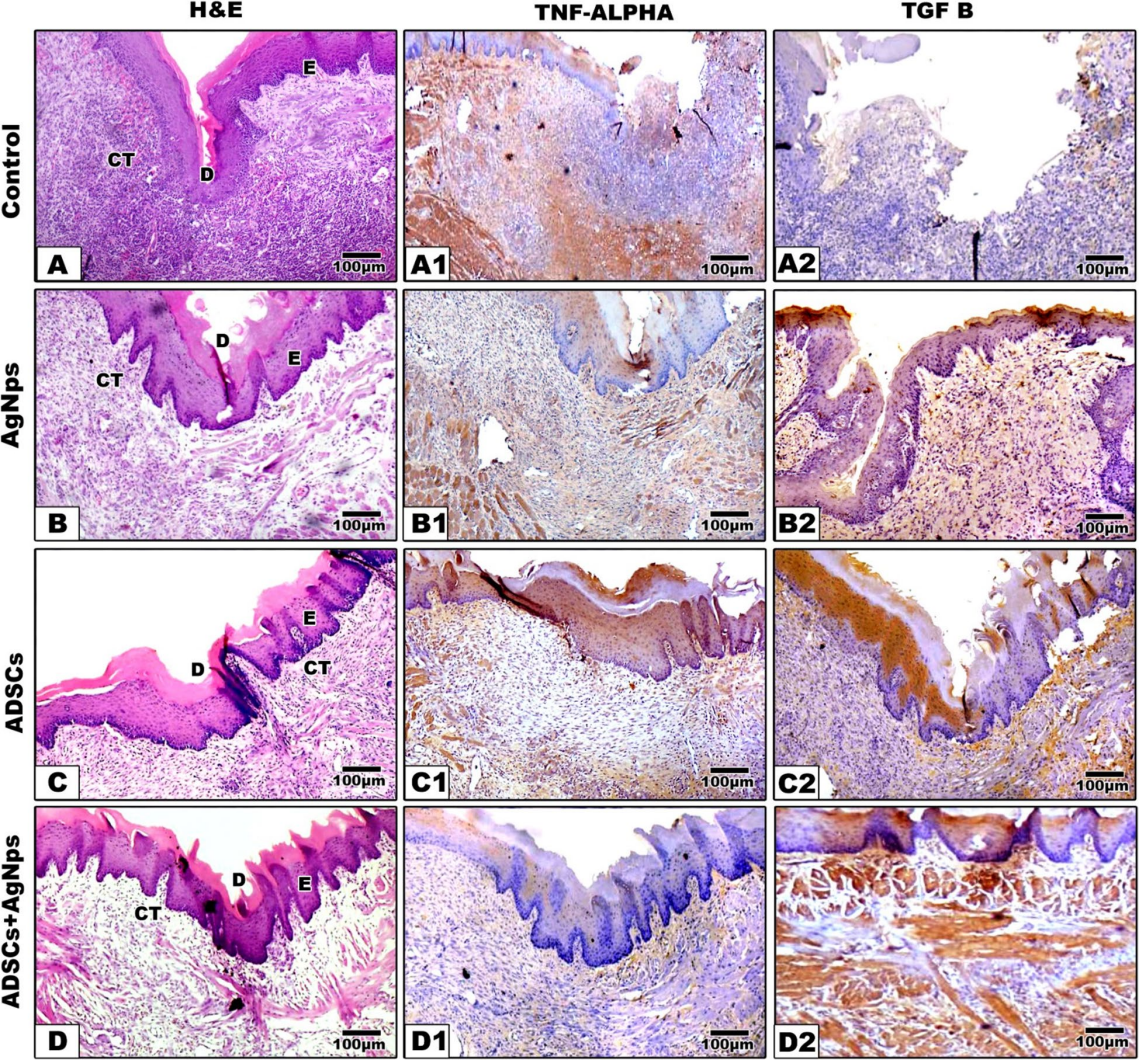
Photomicrographs showing Hematoxylin & Eosin (**A**-**D**), TNF- α (A1-D1) and TGF-β (A2-D2) expression in different treated groups after 7 days. In the controls, the defect left opened filled with granulation tissue with intensive inflammation. In AgNPs, the defect started to close with extensive inflammatory infiltration. In ADSCs, the defect appears to be almost closed with inflammatory cell infiltrate the underlying connective tissue. While group IV (ADSCs + AgNPs) revealed closure of the tongue wound with obvious keratin layer as well as epithelial rete pegs interdigitation with the underlying connective tissue which still infiltrated with inflammatory cells. E: epithelium, CT: connective tissue, M: muscles, D: defect, scale bar = 100 μm

All animals utilized in this investigation lived without experiencing any problems. By the seventh day, there were no signs of infection or necrosis at the defect sites. The defect sites did not exhibit any clinical indications of inflammation, scar formation, or adverse tissue response. After inducing the surgical defect, all of the rats recovered from anesthesia and were able to feed themselves. They also showed no signs of discomfort or infection, such as a red, hot incision or exudate, during the healing phase.

### Histological assessment

*After 4 days*, the control group revealed deep defects covered by keratinized epithelium that did not comprise the distinctive lingual papillae. The underlying connective tissue revealed heavy infiltration by several inflammatory cells with no features of muscle regeneration. In the treated groups, the defects were almost filled with granulation tissue with inflammatory cell infiltrate **(**Fig. 6**)**.

*After 7 days*, the defect boundaries in the control group were still apparent. Newly formed blood vessels and numerous inflammatory cells at wound margins and depth were observed, with no signs of regenerating muscles. In groups II and III, the wound was filled with better-organized granulation tissue with more newly regenerated blood vessels and more organized collagen fibers. Regenerating migrating epithelium was noticed at the lateral wound boundaries with a definite keratin layer. While group IV (ADSCs + AgNPs), a delicate layer of overlying regenerating epithelium was seen at the lateral wound margins, healing was significantly improved, characterized by more regular epithelial and keratin layers of uniform thickness. The connective tissue displayed better-organized collagen bundles and increased vascularity **(**Fig. 7**)**.

### Inflammation (Figs. 6 and 7) (Table 1)

The degree of inflammation was assessed by TNF-alpha, which revealed a reductive trend over time in all groups except the control group. Insignificant differences were determined between either the control, AgNPs and ADSCs groups at 4 days (*P* > *0.05)*, while showing significant difference after 7 days (*P* < *0.05)*. No significant difference was noticed in the inflammatory reaction between ADSCs, AgNPs and ADSCs + AgNPs groups at 4 days (*P* > *0.05)*. While the expression of TNF-alpha was significantly different between the treated and control groups (*P* < 0.05) on day 7. In the control animals, the expression was *(4 days: 4.127 ± 0.10*,* 7 days: 4.208 ± 0.12)*, while the AgNPs group expression was *(4 days: 3.679 ± 0.20*,* 7 days: 2.131 ± 0.07)*. In the ADSCs group, TNF-alpha expression was *(4 days: 3.908 ± 0.40*,* 7 days: 3.347 ± 0.08)*, while the group treated with ADSCs + AgNPs revealed marked reduction in the brown positive expression *(4 days: 3.523 ± 0.40*,* 7 days: 1.463 ± 0.43)*. These outcomes imply that the ADSCs treated with AgNPs might significantly affect the tongue mucosal wound inflammation by reducing TNF-alpha expression, which would become more noticeable after only 7 days.

**Table 1:** Means and standard deviations of Tukey Post-Hoc test of TNF alpha

TNF alpha				
Groups *Time*	**Control**	**AgNPs**	**ADSCs**	**ADSCs + AgNPs**
Mean ± SD at 4 days	4.127 ± 0.107^A^	3.679 ± 0.201^ABC^	3.908 ± 0.4^AB^	3.523 ± 0.401^BC^
Mean ± SD at 7 days	4.208 ± 0.127^A^	2.131 ± 0.075^D^	3.347 ± 0.081^C^	1.463 ± 0.434^E^

Means with different superscript letters are significantly different at *P* < 0.05

### Re-epithelialization (Figs. 6 and 7) (Table 2)

The degree of re-epithelialization was assessed by TGF-β, which demonstrated an ascending trend over time in all groups. Insignificant differences were determined between 4 and 7 days among the tested groups (*P* > 0.05). However, TGF-β expression in the group treated with ADSCs + AgNPs seems to be significantly higher in comparison to either control or treated groups in the two tested time points. In the groups treated with ADSCs + AgNPs after 4 days, the TGF-β expression was *(6.545 ± 0.38)* and after 7 days *(7.053 ± 0.49)*. While in ADSCs and AgNPs treated groups, the degree of re‐epithelialization after 4 days *(ADSCs: 4.362 ± 0.17)*,* (AgNPs: 2.534 ± 0.51)* and after 7 days *(ADSCs: 4.815 ± 0.14)*,* (AgNPs: 2.834 ± 0.11)*. Noteworthy that the highest degree of re‐epithelialization was noticed in the ADSCs + AgNPs group after 7 days *(7.053 ± 0.49)*.

**Table 2:** Means and standard deviations of Tukey Post-Hoc test of TGF-β

TGF-β				
Groups *Time*	**Control**	**AgNPs**	**ADSCs**	**ADSCs + AgNPs**
Mean ± SD at 4 days	1.421 ± 0.263^D^	2.534 ± 0.512^C^	4.362 ± 0.179^B^	6.545 ± 0.389^A^
Mean ± SD at 7 days	1.434 ± 0.246^D^	2.834 ± 0.117^C^	4.815 ± 0.142^B^	7.0536 ± 0.493^A^

Means with different superscript letters are significantly different at *P* < 0.05

## Discussion

The use of nanotechnology in several fields has made it more significant in recent years. AgNPs are one of the fastest-growing products because of their improved antibacterial action at the nanoscale level. They are widely utilized in wound dressings to aid in the healing process as they have a strong antibacterial property [20–23]. Due to their prolonged ability to release Ag ions and their concentration-dependent cytotoxicity, these nanoparticles offer a lot of potential for application in wound healing [24, 25]. However, despite their growing number of applications, more research is still needed to fully understand their biology due to the many controversial discoveries on their safety that have been published [26].

In this study it was crucial to evaluate the cytotoxicity of various AgNPs concentrations on ADSCs because cytotoxicity is a major concern when assessing the biocompatibility of certain materials. Interestingly, after 72 h of incubation, there was a gradual decrease in the number of viable cells as the concentration of AgNPs increased [27]. Thus, 2.5 µg/ml concentration was chosen as it showed the highest percentage of viable cell number in comparison to all other tested concentrations. The discrepancy between published articles regarding AgNPs safe concentration is likely due to the various AgNPs properties, including size, coating type, and surface chemistry, which can affect biological outcomes like cytotoxicity, intracellular localization, and nanoparticle uptake. Changing any one of these parameters can have significant consequences [28, 29].

In this study, we used the labelling technique to confirm the role of ADSCs in the healing process of the tongue [12]. Prior to injection, the ADSCs were labelled with iron oxide and Prussian blue dye was used to detect ADSCs homing into the defect area in order to identify the presence of iron oxide in the epithelium or lamina propria. This confirmed that the ADSCs were either fully or largely responsible for the healing process. Granular purple or blue residues of iron-labelled ADSCs were found in both the ADSCs group and the ADSCs + AgNPs group.

Hemostasis, inflammation, proliferation, and maturation are the four primary overlapping phases of wound healing that occur in various tissue types [30]. Dense inflammatory infiltrate was observed at the margins and bases of the wounds at days 4 and 7 after surgery. This conforms to the fact that the inflammatory phase begins as early as 24–48 h and can last up to a week after injury, where inflammatory cells are recruited as a result of the chemokine secretion at the injury site [31].

In all groups in our study, after 4 days, granulation tissue began to grow in the wound site to varying degrees. By 7 days, the tissue was more organized and vascularized. This agrees with Des Jardins-Park et al., who clarified that during the proliferative stage of healing, in response to the released signaling molecules, the extracellular matrix is replaced by a vascular stroma that leads to granulation tissue formation, and re-epithelialization starts at the wound edges [31]. However, partially healed wounds were visible in the control group, while the AgNPs group displayed a significant reduction in defect and hastening of wound healing compared to the controls [32]. In the same line, Abdel Aal et al., stated that treatment with AgNPs resulted in diminished numbers of inflammatory cells secondary to suppression of bacterial replication and reduction of proinflammatory cytokines, in particular TNF-alpha, and as a result, reduced the formation of matrix metalloproteinases with a subsequent reduction in wound inflammation [33, 34]. Metal ions are generally thought to have a significant effect on the activation of redox-sensitive transcription factors like NF-κB or AP-1 [35]. These transcription factors are involved in inflammatory responses and are important for processes like differentiation and cell growth [36]. Ag-mediated oxidative stress can cause the nuclear translocation of NF-κB, which controls pro- and anti-inflammatory genes [37–39] which is thought to be a common mechanism for Ag-induced effects.

The ADSCs treated group showed good healing potential through the tested time points, with well-organized granulation tissue filling the defects after 7 days [40–42]. In the tested group where ADSCs treated with AgNPs, better wound healing with further reduction in the defect size after 7 days was noticed; however, Sengstock et al., reported that internalization of AgNPs into ADSCs had a significant effect on different forms of cellular functions [43].

It has been confirmed that high and persistent inflammation may cause wounds to heal more slowly. Thus, TNF-alpha immunostaining was utilized in this context to evaluate the inflammatory response of the wounded tissues since it is a frequently used inflammatory marker and one of the cytokines implicated in immune responses during inflammation [44]. Our results showed decreased inflammatory reaction in the experimentally treated groups after 7 days as compared to the control [45]. It has been proved that MSCs aid in the removal of infection from wounds by directly secreting antimicrobial substances and encouraging immune cell phagocytosis. When managing chronic wounds, in which a high degree of inflammation impedes healing, MSCs’ capacity to facilitate the shift from the inflammatory to the proliferative phase is very important. By expressing growth factors like TGF-ß to encourage granulation and epithelialization, MSCs also support the proliferative phase. Finally, MSCs promote organized extracellular matrix deposition, which controls remodeling of the healed wound [46]. Taken together, this could explain the superior healing effect of ADSCs treated with AgNPs in comparison to other treated groups after only 7 days of treatment. However, further studies are needed to test the impact of this combination over longer durations.

### Limitations of the study

It is essential to acknowledge the limitations of the current study. Since only one cell line was studied, variations in cell line responses may cause the cytotoxicity findings to vary. The study’s short durations, 4 and 7 days, might not have taken into consideration the prolonged durations. To provide a thorough assessment, additional research should consider a variety of cell lines, exposure durations, and concentrations.

## Conclusion

Few treatments presently combine the therapeutic benefits of MSCs, which are particularly important for wounds that are difficult to heal, despite the fact that there are many products available to treat wounds. Numerous initiatives are underway to create innovative bioengineered wound-healing solutions, and taking into account the function of MSCs in conjunction with AgNPs illuminates a new era of promise for wound healing.

## Data Availability

The study’s data can be obtained by contacting the corresponding author.
